# Virtual World Currency Value Fluctuation Prediction System Based on User Sentiment Analysis

**DOI:** 10.1371/journal.pone.0132944

**Published:** 2015-08-04

**Authors:** Young Bin Kim, Sang Hyeok Lee, Shin Jin Kang, Myung Jin Choi, Jung Lee, Chang Hun Kim

**Affiliations:** 1 Interdisciplinary Program in Visual Information Processing, Korea University, Seoul, Korea; 2 Department of Computer and Radio Communications Engineering, Korea University, Seoul, Korea; 3 School of Games, Hongik University, Seoul, Korea; East China University of Science and Technology, CHINA

## Abstract

In this paper, we present a method for predicting the value of virtual currencies used in virtual gaming environments that support multiple users, such as massively multiplayer online role-playing games (MMORPGs). Predicting virtual currency values in a virtual gaming environment has rarely been explored; it is difficult to apply real-world methods for predicting fluctuating currency values or shares to the virtual gaming world on account of differences in domains between the two worlds. To address this issue, we herein predict virtual currency value fluctuations by collecting user opinion data from a virtual community and analyzing user sentiments or emotions from the opinion data. The proposed method is straightforward and applicable to predicting virtual currencies as well as to gaming environments, including MMORPGs. We test the proposed method using large-scale MMORPGs and demonstrate that virtual currencies can be effectively and efficiently predicted with it.

## Introduction

Virtual economies have emerged through interactions among users in virtual worlds. A virtual economy is primarily intended to foster users’ increased enjoyment of the virtual environment. In some cases, the virtual economy lends itself to real economic purposes [1, 2]. Virtual economies have been evident in virtual reality social services or generally in multiplayer virtual reality games in which multiple users interact with one another. For example, in the virtual world of Second Life, a virtual currency called Linden Dollars is used. This currency is used within the virtual environment for buying and selling of houses, clothes, and other items made by users. In the gaming environment of World of Warcraft, which is a massively multiplayer online role-playing game (MMORPG), the virtual currency referred to as Gold is used to buy items for gaming and for other transactions among users.

In addition, the trend of exchanging real money for virtual currencies has been increasing [3]. Several users in Second Life have been trading Linden Dollars for real money or the Bitcoin virtual currency [4]. On eBay, some users have been selling for cash the virtual currencies used in MMORPGs, such as World of Warcraft and EVE Online. The percentage of such users has been steadily growing [5, 6]. Moreover, the size of this market has been increasing and is expected to grow further [4]. In this context, diverse research on the relevant market is underway [1, 3, 5–11]. Nevertheless, previous studies primarily focus on the market formed by virtual currencies with minimal consideration of the values of virtual currencies used in the market. Hence, a means of predicting the values of virtual currencies and acquiring insights into general market trends is needed.

Many studies have been conducted for real economic purposes. Particularly, studies on stock price prediction techniques date to the beginnings of stock trading [12–18]. Since then, some researchers have predicted stock prices based on neural networks [12, 14], while others have applied learning through support vector machines (SVMs) [15]. Data used for predictions is predominantly grounded in financial news [13, 17]. Recently, web-based data has been used to analyze the stock market [16, 18]. Bollen et al. [16] analyzed tweets on Twitter to deduce the mood of users and to thereby predict the stock market. Nevertheless, few studies have evaluated and predicted virtual market trends. Numerous users have engaged in virtual currency transactions and have formed a large market; therefore, predicting the values of virtual currencies has proved to be increasingly important in connection with transactions of real goods. Predicting the values of virtual currencies can help traders engage in rational transactions, while enabling developers who control virtual worlds to identify and rectify the problems of the respective virtual economies [19, 20].

The virtual world is gradually expanding and becoming increasingly more complex. The existence of big data from such an environment can be used for research on social theories in large-scale virtual populations [21, 22] from different perspectives [23–27]. Research on the structure and dynamic evolution of social networks in virtual worlds has been based on various methods and produced significant results [28–33]. Our present objective is based on the assumption that the virtual currency used among users in mutual interactions fluctuates. In our study, we reference the properties of virtual worlds as well as data on the voluntarily provided opinions of the users.

Analysis of online user opinion data has been underway in many fields. Users express their opinions and sentiments and review products in online social networks [34–36]. Thus, user data has become a valuable resource for research [34–37]. Recently, real-time user data on Twitter has been used to predict events, such as earthquakes [38, 39]. Many researchers refer to Twitter as well as to social networking sites, such as Facebook and LinkedIn [40, 41]. In addition, deducing the emotions or sentiments from postings on social networking sites has been valuable in many aspects [42–46]. For example, some researchers have developed and used common sentiment lexicons for analyzing general emotions [47, 48], while others have employed target-specific approaches to film blogs or wine reviews for sentiment analysis or opinion mining [49, 50]. Users’ reactions and comments on social networking sites serve as significant indicators of the users’ momentary sentiments, thereby exerting significant effects on virtual environments [51]. In various online communities, including social networking sites, discussions of virtual worlds and the revealing of users’ states of mind have been evident [35, 52].

In this paper, we analyze user sentiments in popular virtual communities on the Internet to predict the fluctuations and values of virtual currencies. The proposed method for analyzing online communities is based on the Bollen et al. [16] analysis of tweets on Twitter to deduce the mood of users and to thereby predict the stock market. Datasets are selected for a virtual environment or larger virtual world. Moreover, the proposed sentiment analysis model is not dependent on a specific market as conventional models are; rather, it is easy to use and fits the features of a virtual world.

Virtual currencies in virtual worlds, such as those in online gaming, differ from real money because the former operate on a specific economic system that supports an environment for perfect market competition [53]. In such a system, multiple producers and consumers exist, while consumers do not affect price fluctuations—considering that all items are replaceable, no limitations exist in altering the production of goods, all economic subjects have perfect information, and no asymmetry of information exists [54]. Accordingly, fewer variables for currency value fluctuations exist compared to a real currency system; value fluctuations are clear and intuitive. Thus, instead of an integrated collection of data, selecting datasets from the most prominent online community in a virtual world facilitates more accurate prediction of currency value fluctuations. Furthermore, most suppliers and consumers of virtual currencies are not rational profit maximizers; instead, they are sentimental/emotional game players seeking enjoyment, and analysis of their sentiments is therefore important. The proposed method is designed to meet the characteristics of virtual worlds. It efficiently predicts virtual currencies with a simple sentiment analysis model and narrow data extraction.

The proposed method is general and thus applicable to predicting a range of virtual currencies. The proposed system is basically divided into two parts: learning and evaluation. First, for the learning stage, community data that is relevant to the virtual world under analysis is crawled and classified based on dates. Next, based on user reactions that are collected daily, the sentiment analysis is performed. From that point, machine learning is performed by connecting users’ states of mind (sentiments), numbers of postings, and positive reactions to postings on a specific date with virtual currency fluctuations. In the evaluation stage, the values of the virtual currencies are predicted based on the model learned in the previous stage. This method is proven to predict the values of currencies used in several virtual worlds.

## Methods

### System Overview

**Fig 1:**
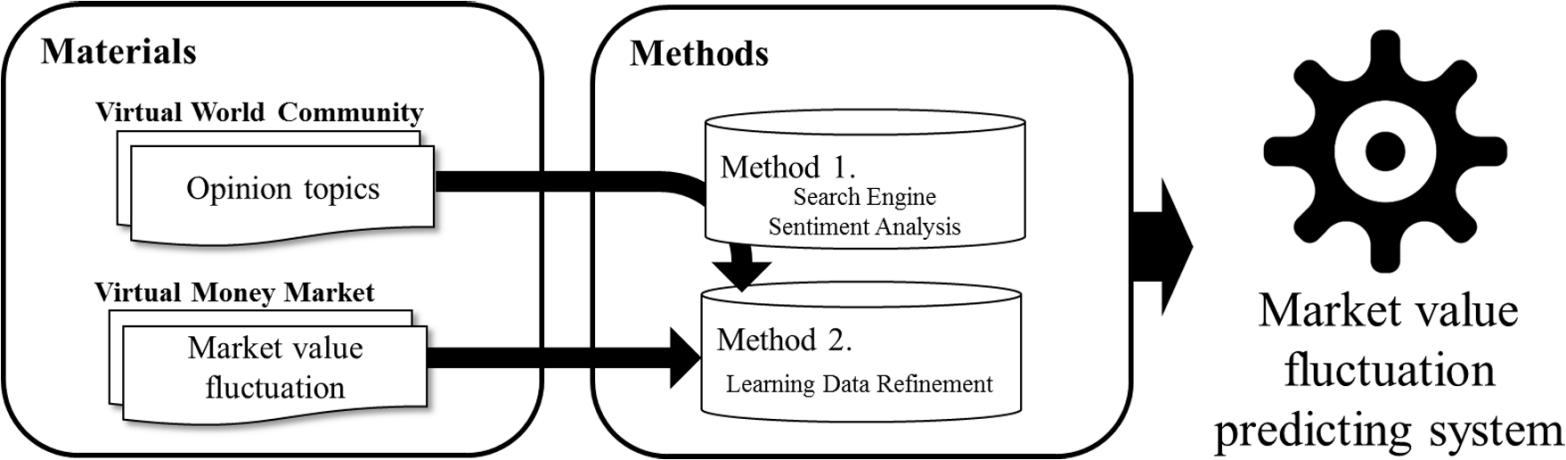
System overview.

The proposed system is comprised of two basic parts, as mentioned above. One part involves learning the rise or fall of currencies based on user data from social networks or communities. The other part involves an evaluation based on the user data and learned model (see Fig 1).

The system learning component follows the steps below to generate the learning model.

1. Crawl user opinion data that is relevant to the virtual world for analysis of a given period of time.
2. Analyze user sentiments based on the obtained user opinion data.
3. The Granger causality test is based on the preselection of sentiments that are highly relevant to value fluctuation.
4. Perform learning based on the obtained opinion data with the evaluation of user sentiments and virtual currency fluctuation data.

The system evaluation component follows the steps below to predict virtual currency value fluctuations and to evaluate the model.

1. Crawl user opinion data that is relevant to the virtual world for use in the evaluation.
2. Analyze user sentiments based on the obtained user opinion data.
3. Evaluate the precision of forecasting the following day’s virtual currency fluctuations based on the learning model constructed in the learning part.

In the next section, the data and algorithm used in each of the two system components are described.

### Data Crawling and Refinement

Any data that is randomly extracted from the virtual world of interest is minimally significant. Therefore, it is necessary to sort the data for collection. For the opinion data, a community with the largest number of daily discussion postings is chosen from online communities associated with the given virtual world. For the market data, a service for collecting the values of virtual worlds and providing the statistics is chosen. To ensure the precision of the market data itself, the data should come from the internal systems of the respective virtual worlds (auction houses or bazaars) or from any sellers dealing in virtual currencies in actual cash. The data chosen for the present study fulfills these two conditions.

Crawling is intended to collect user opinions. Here, an HTML crawler is customized for the web structures of the given online community and the service that provides the market data; it extracts user opinion data for each set period. The extracted opinions and market data are grouped into sets based on the period of choice (normally a 24-hour period). As per the sets, the topics, contents, points of time for posting, comment counts, and view counts of the opinion data are saved. The current market value and fluctuation compared with those of the previous day are saved as the market data.

Upon completion of crawling, the collected data requires refinements because it still contains considerable garbage or redundant information and is not sufficiently uniform. To delete the garbage or redundant data, many spam filtering techniques have been explored [55–59]. Here, any opinion data with more than three repetitions of content per day is handled as garbage. In addition, comment and view counts for a topic are normalized with the counts of discussion postings on the same day.

### Sentiment Analysis of User Opinion Data

The collected user opinion data is used for sentiment analysis. First, user opinion data is extracted based on the significant parts of speech. To this end, in English-speaking regions, the Stanford Parser [60] is used to extract the part of speech for each word, whereas the tools of Monroe et al. [61], and Tseng et al. [62] or Chang et al. [63], are used in Arabic- and Chinese-speaking worlds, respectively, to extract words of desired parts of speech. To note, parsers vary with the language used for data extraction. Here, to analyze the parts of speech in English sentences, the Stanford Log-linear Part-Of-Speech Tagger [64] is used to sort out words of significant parts of speech—i.e., verbs, adjectives, and adverbs—for sentiment analysis. The words of these parts of speech are saved for sentiment analysis. Modifiers, nouns, and postpositions that enrich the meaning of a sentence are not sorted out because they are most likely neutral words; accordingly, this approach provides the precision of analysis. Moreover, sentences in Internet community postings are often marked by incorrect punctuation, spelling, or word order. Thus, including too many parts of speech in the analysis can cause adverse effects and consequently obscure the real meanings of sentences.

In this paper, we present a straightforward method for sentiment analysis. Human emotions are too complicated to sort out. Hence, Plutchik’s research on human emotions [65] is used. Specifically, the Plutchik wheel of emotions [65] used here is a reasonable model that represents combinations of solid, relative, and complementary aspects of human emotions. Eight primary emotions—joy, trust, fear, surprise, sadness, disgust, anger, and anticipation—are applied as the criteria for the present analysis. The wheel of emotions is employed as criteria because it is composed of four pairs of ambivalent emotions with contrary concepts; moreover, the combinations of these eight emotions can help deduce more complicated emotions. In other words, instead of a dichotomic analysis of emotions as positive or negative ones, it is possible to build a profound and scalable model for sentiment analysis. Plutchik’s model has been used for sentiment analysis [66, 67], artificial neural networks [68], decision making models [69], and multi-modal convergence [70].

In terms of the criteria for parts of speech and emotions, a search engine is used for the sentiment analysis. The sentiment analysis consists of four phases in total. In the first phase, the opinion data (*Opi*) are split into words using the part-of-speech tagger [64]; each word is matched to an appropriate part of speech (*Pos*). When a word’s part of speech corresponds to ‘a desired part of speech (*Pos*: verb, adjective and adverb defined above),’ the word is transferred to the second phase. In the second phase, the target word that has passed the first phase undergoes a search query together with ‘emotive words (*Emo*: eight emotions above defined).’ For example, if the target word is ‘greedy’ and the emotive word is ‘joy,’ the query becomes ‘greedy joy.’ To compare each word with the eight emotive words, a total of eight queries are performed. In the third phase, the findings from the query analysis are used to yield a ‘Word_R,’ which is a set of relevance figures between the target and emotive words. Word_R is defined as the mean of minimum values of numbers of blanks between the target word and emotive word within the result pages.

**Fig 2:**
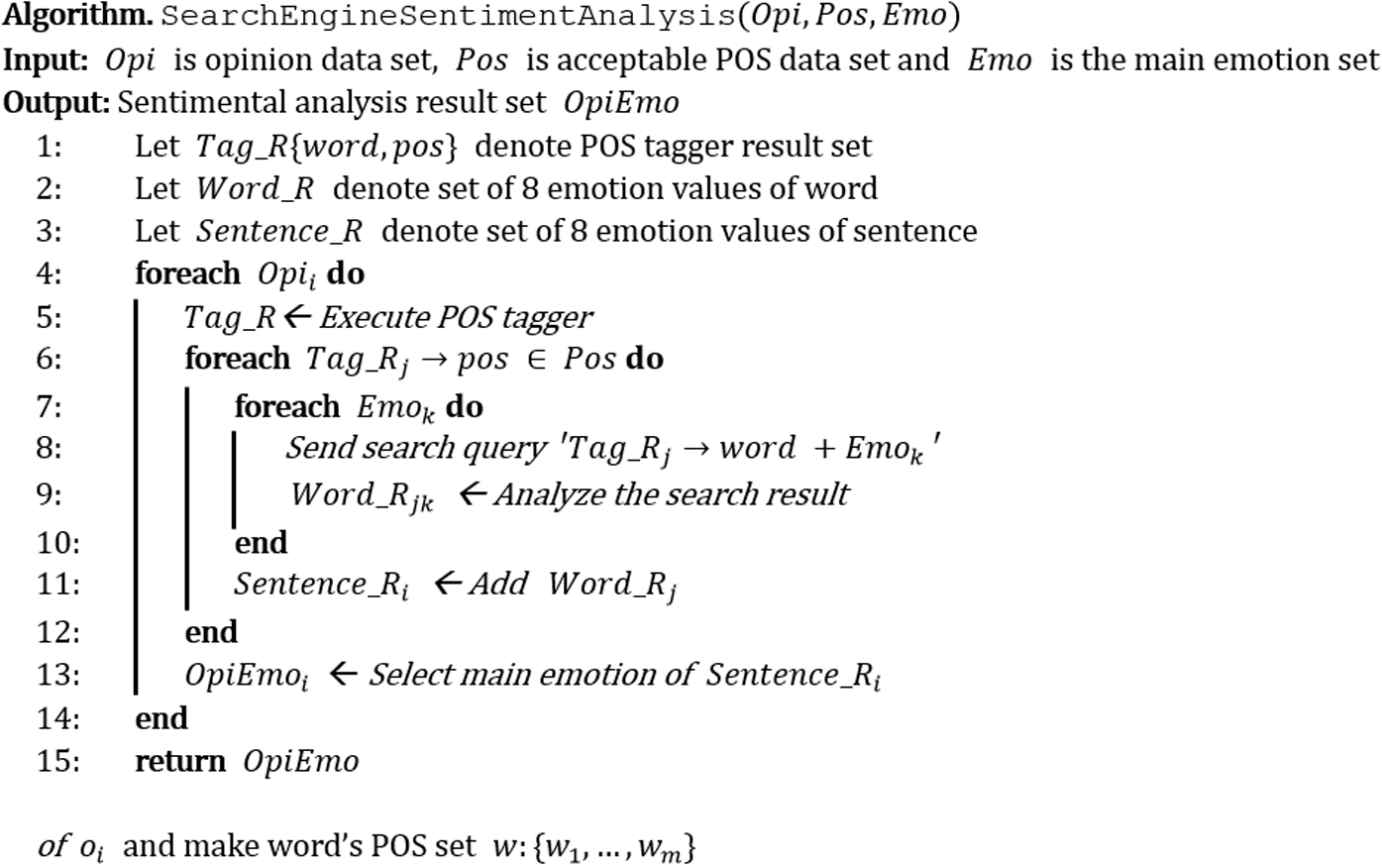
Algorithm of the search engine sentiment analysis method.

In the fourth phase, the Word_Rs representing the relevance between the target and emotive words collectively comprise the ‘Sentence_R’ set, which represents the relations between a given sentence and its emotive words. Among the eight emotion values stored in Sentence_R, emotion, which has the smallest value, is defined as the primary emotion of the opinion data. This is because the defined emotion values—the mean distance between the target word and emotion word—are highly associated if the emotion value decreases. If the number of blanks is zero in all eight emotions, the opinion data is defined as ‘neutral.’ If this type of four-phase emotion analysis is completed, the major emotions of the target opinion data can be designated as one of nine emotions. Fig 2 summarizes this process.

The strength of the proposed sentiment analysis method using search engines is that it is optimized for mood analysis in multiple virtual worlds. Users in virtual world communities can share their current opinions online; therefore, the collection of countless online documents by search engines provides an efficient means of viewing the statuses of virtual worlds. Indeed, notices of virtual world developers, updates, user information sharing, and socializing are all online and can be collected by search engines. The present analysis of documents found by search engines enables a simple numeric analysis of emotions. Furthermore, a sentiment lexicon can be implemented by repeating the aforementioned process while saving data.

Use of search engines for sentiment analysis minimally differs from that of ordinary search engines. That is, the relevance between the search terms and emotive words is analyzed by combining these words and sending a query to obtain the search results. Hence, it is possible to freely analyze the relevance between countless topics and emotions with nothing but word combinations in queries. It is additionally possible to extract sentiment data for specific topics by adding search words, and to use search engines for general purposes in diverse languages by simply using different languages in the queries (see the sentiment analysis example in Table 1).

**Table 1:** Sentiment analysis example.

Emotional Criteria	Example topic sentences
Neutral	“Warlords of Draenor Launch Update: 1:40 p.m.” / “Message from J. Allen Brack” / “Character creation” / “Rare spawn chance for Huolon”
Joy	“Thanks, Blizzard!” / “Mists of Pandaria 10 Day Free Trial” / “Anniversary Event Extension” / “Blizzard says it's ok to be a jerk”
Trust	“Female Gamers Group want a Safe Space.” / “Alliance needs a revamp, any suggestions?” / “Face it Alliance, you are EVIL.”
Fear	“Help control Blood Elf population!” / “What's going on with the WoW Armory?” / “No ghost flying in pandaria?” / “Please remove LFR.”
Surprise	“Whats happened to WoW?” / “Why no-flying in Draenor is a good thing!” / “What Happened to Blizz's Customer Support?!”
Sadness	“Server Maintenance soo. . .rate that xmog!” / “World Server Down” / “The Iron Horde has already failed” / “Sad to see so many leaving”
Anger	“Every US server is LOCKED” / “Curse client not connecting to the internet?” / “Players refusing to use abilities” / “Fix the Vote to Kick”
Anticipation	“The new models aren't final, right?” / “Least/Most popular class?” / “New Race Idea: Gargoyles!” / “Your most memorable WoW moment”

**Fig 3:**
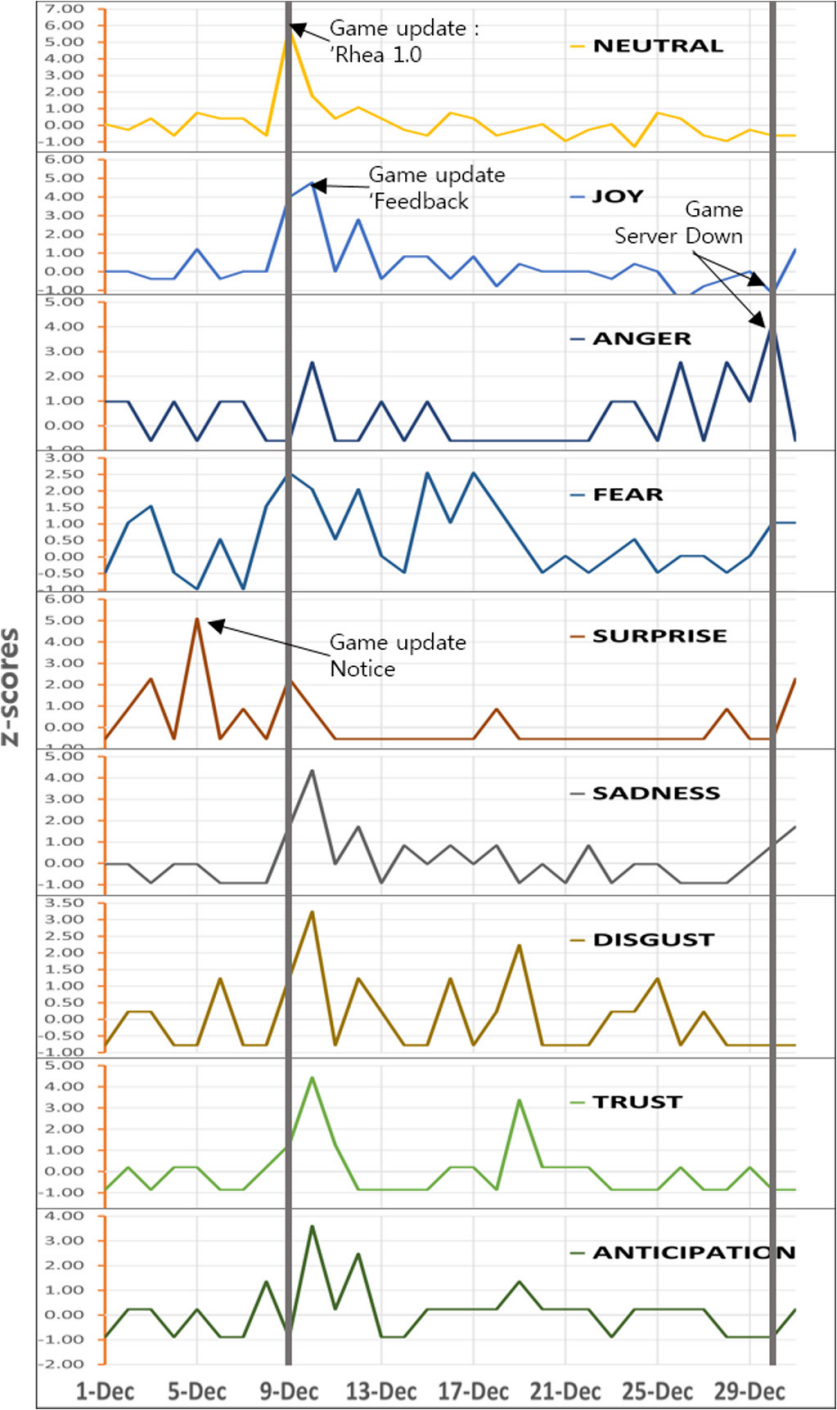
Tracking emotional relevance with the EVE Online actual update.

To compare the mutual interactions between the sentimental analysis results and events happening in the virtual world, we normalized values to z-scores on the basis of a local mean and standard deviation to the degree of the sliding window of *k* days before and after a particular date. Such normalization was established so that all time series fluctuate around a zero mean and are expressed on a scale of one standard deviation. We executed sentimental analysis for the month-long data throughout December 2014 for the EVE Online community[71] and normalized the result value as a z-score. We examined the relevance with the date when the actual update occurred. We identified the occurrence of general sentiment changes in the community for events that occurred in the virtual world, such as game update notices, major updates, and virtual world server-down notifications (see Fig 3.).

#### Configuration and Evaluation of the Prediction Model via Support Vector Machine Learning

User sentiment values change in significance according to the occurrence of particular events. However, the association between the sentiment values and market value fluctuation must be observed; moreover, appropriate emotions must be used in the prediction model to produce positive results. We therefore applied the econometric technique of Granger causality analysis [72]. In this paper, the daily time series produced by sentiment analysis was normalized to the z-score. In addition, linear interpolation was executed so there would be no day for which the market value was missing in terms of the virtual world currency value. Moreover, a different market value on the previous day was normalized to the z-score for executing the Granger causality test. We did not test the actual causation; rather, we examined whether one time series had predictive information about the other.

**Fig 4:**
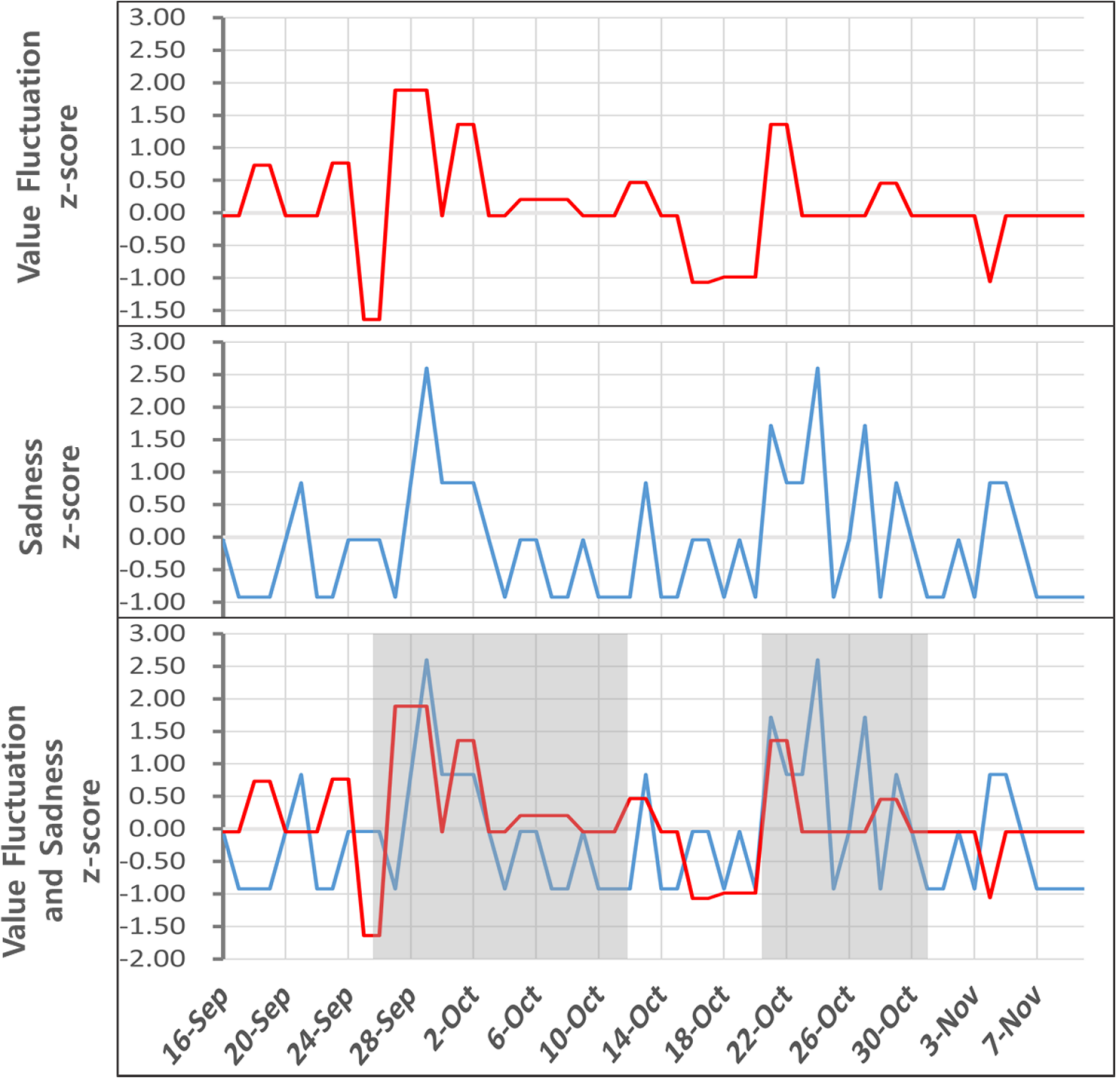
Bottom graph shows the overlap of z-scores of price fluctuation and emotion data.

Data in Tables 2–4 and Fig 4 enable the Granger causality test results to be compared across virtual worlds. We identified differences in high sentiments for virtual currencies and Granger causality results across virtual worlds. This approach was based on the study in [73], which preselected data groups with high causality for learning while excluding the data group with a small amount of causality. That study utilized the Granger causality test to execute learning based on sentiment data that displays high causality.

**Table 2:** Statistical significance (*p-*values) of bivariate Granger causality correlation for the Bitcoin experiment.

Lag	Emotion Data								
	Neutral	Joy	Trust	Fear	Surprise	Sadness	Disgust	Anger	Anticipa-tion
1 day	0.110	0.551	0.056	0.0055*	0.770	0.193	0.578	0.088	0.239
2 days	0.197	0.687	0.0031*	0.0036*	0.836	0.483	0.838	0.073	0.285
3 days	0.270	0.617	0.0002*	0.0004*	0.0171*	0.422	0.788	0.115	0.310
4 days	0.472	0.661	0.0007*	0.0016*	0.0231*	0.496	0.865	0.0396*	0.117
5 days	0.158	0.609	0.0007*	0.0020*	0.0076*	0.588	0.930	0.067	0.146
6 days	0.205	0.791	0.0003*	0.0012*	0.0015*	0.089	0.769	0.112	0.158
7 days	0.157	0.869	0.0003*	0.00002*	0.0027*	0.130	0.837	0.159	0.101

(*p*-value < 0.05: *)

**Table 3:** Statistical significance (*p-*values) of bivariate Granger causality correlation for the EVE Online experiment.

Lag	Emotion Data								
	Neutral	Joy	Trust	Fear	Surprise	Sadness	Disgust	Anger	Anticipa-tion
1 day	0.220	0.835	0.278	0.413	0.844	0.655	0.882	0.498	0.149
2 days	0.293	0.712	0.539	0.691	0.659	0.862	0.376	0.805	0.110
3 days	0.426	0.644	0.802	0.534	0.832	0.0088*	0.264	0.550	0.0298*
4 days	0.647	0.834	0.959	0.433	0.901	0.0139*	0.516	0.763	0.0421*
5 days	0.625	0.812	0.515	0.483	0.889	0.0197*	0.754	0.077	0.0403*
6 days	0.819	0.927	0.326	0.514	0.937	0.0323*	0.509	0.183	0.142
7 days	0.832	0.924	0.432	0.445	0.963	0.0482*	0.673	0.135	0.265

(*p*-value < 0.05: *)

**Table 4:** Statistical significance (*p-*values) of bivariate Granger causality correlation for the World of Warcraft experiment.

Lag	Emotional Data								
	Neutral	Joy	Trust	Fear	Surprise	Sadness	Disgust	Anger	Anticipa-tion
1 day	0.054	0.0264*	0.113	0.0399*	0.0190*	0.0168*	0.134	0.054	0.054
2 days	0.054	0.0287*	0.137	0.058	0.051	0.0427*	0.178	0.147	0.0048*
3 days	0.107	0.079	0.273	0.121	0.120	0.115	0.261	0.322	0.0159*
4 days	0.127	0.074	0.278	0.119	0.193	0.129	0.324	0.309	0.0209*
5 days	0.213	0.133	0.391	0.192	0.238	0.214	0.464	0.445	0.0441*
6 days	0.231	0.153	0.401	0.261	0.316	0.249	0.457	0.489	0.060
7 days	0.325	0.215	0.495	0.337	0.317	0.345	0.565	0.590	0.094

(*p*-value < 0.05: *)

The machine learning performed in this study is based on the aforementioned database. For the machine learning, it is necessary to sort out date-specific user sentiment data sets. The following are used as input data: each emotion value of the data-specific opinion data previously obtained and the respective number of user posts, inquiries, and comments. In the case of emotion values, as the formula (1), normalized emotion values to z-scores are used on the basis of a mean and standard deviation. In certain date t, z-score of emotion data $E_{t}$, denoted $Z_{E_{t}}$, is defined as:

$$Z_{E_{t}}=\frac{E_{t}-\bar{x}\left(E\right)}{\sigma \left(E\right)}$$ (1)

where $\bar{x}\left(E\right)$ and σ(E) represent the mean and standard deviation of the emotion data for every date. In terms of the respective numbers of user posts, inquiries, and comments, the absolute values are not used as they currently exist; rather, the number of posts on the previous day, number of inquiries, and the relative change value from the number of comments are used. This is simply compared with the number of posts, inquiries, and comments on the previous day to obtain the variance value. An example of applicable input data is shown in Table 5.

**Table 5:** Machine learning data set example.

Data Class	Date	Emotion Data									Topic Data		
		Neutral Topic	Joy Topic	Trust Topic	Fear Topic	Surprise Topic	Sadness Topic	Disgust Topic	Anger Topic	Anticipation Topic	Number of Topics	Sum of Comments	Sum of Inquiries
Crawled Raw Data	Dec 23	a	b	c	d	e	f	g	h	i	x	y	z
	Dec 24	A	B	C	D	E	F	G	H	I	X	Y	Z
Input Learning Data	Dec 24, 2013	$Z_{{\text{A}}_{t}}$	$Z_{{\text{B}}_{t}}$	$Z_{{\text{C}}_{t}}$	$Z_{{\text{D}}_{t}}$	$Z_{{\text{E}}_{t}}$	$Z_{{\text{F}}_{t}}$	$Z_{{\text{G}}_{t}}$	$Z_{{\text{H}}_{t}}$	$Z_{{\text{I}}_{t}}$	X − x	Y − y	Z − z

With regard to the classification value for learning, the fluctuation in the market price is used for predicting the market price. Compared to the market price data on the previous day, the price data are divided into two classes: one for when the market price increases; the other for when the market price decreases. It is then used as the classification value. The relation between the input data and fluctuation in the market price is sometimes immediately influential; at other times, it is influential after a specific amount of time has passed. In this study, we therefore create models at intervals from one day through seven days in terms of the relation between the input data and market price fluctuation. In case of the emotion value, the number of posts relating to the nine emotions is divided by the total number of posts for sentiments with high causality.

Through the Granger causality test conducted above, we determined that the lag with a high relevance with sentiment data and the market value is between one to seven days. Instead of trying to make assumptions by searching for one accurate model, we strived to execute learning or dates with a high relevance and to confirm the result. Therefore, we selected a model that shows good results among those seven models; discovery of the exact point of such a relation is not the focus. The differences among the results from using the applicable model are discussed in the next section. Moreover, the SVM [74], used for learning and evaluation, is adapted for the present system using a Library for Support Vector Machines (LIBSVM) [75].

Upon completion of the model through learning for evaluating the virtual currency fluctuations based on current user opinion data, the virtual currency fluctuations are predicted using the model. In the following section, we present our analysis of the currencies in existing virtual worlds and application of several methods for evaluation. Accordingly, we account for how the system predicts the results.

## Results

### Experimental Design

We performed the experiment in two ways. First, the proposed method was applied to two well-known virtual currencies for games to predict their values based on real money. Second, the value of Bitcoin, which is a major virtual currency used in everyday life, is predicted in values of real money.

The experimental process was the same as the system process described earlier. First, each virtual world’s opinion data and market data were collected. Then, the sentiment analysis using search engines was performed with the opinion data. The analysis findings were gathered to generate a learning model using the SVM based on the fluctuating market prices of gaming currencies traded in cash. From that point, the prediction system was validated.

In the first experiment, virtual currencies used in two MMORPGs—EVE Online, released in 2002 by CCP Games, and World of Warcraft, released in 2004 by Blizzard Entertainment—were selected for the target currencies for analysis. Both games are popular and have large-scale virtual economies, which many researchers have used for analyzing virtual economies in the gaming world [5, 6]. Of all MMORPGs and virtual worlds, World of Warcraft has the largest subscriber base (more than 10 million) as of November 2014 [76]. Of virtual currency fluctuations, both games show the largest scales; therefore, in this study, we predict the values of virtual currencies in these games.

The other experiment that we conducted addressed the prediction of fluctuations of Bitcoin. This virtual currency is comparable to those analyzed herein; however, it is not used in virtual worlds. Consequently, it is more relevant to real currencies than to virtual gaming currencies. The cash transaction market for Bitcoin outweighs that for World of Warcraft. Here, we applied to Bitcoin the same algorithm that we applied to Virtual World currencies for prediction. It was assumed that Bitcoin is significant for analysis because transactions with actual money are more frequent than those with virtual money within a game. Table 6 outlines the arrangement of market data and opinion data that were gathered.

**Table 6:** Crawled data summary.

Target Virtual Currencies	Market Prices			Opinion Topics		
	Crawling Source	Crawling Boundary	Data Volume(days)	Crawling Source	Crawling Boundary	Data Volume(threads)
EVE Online	mmobux	Jul. 22, 2014~ Jan. 22, 2015	77	EVE Online Forums	Jul. 22, 2014~ Jan. 22, 2015	4,587
World of Warcraft	mmobux	Jul. 22, 2014~ Jan. 19, 2015	74	World of Warcraft, official US site	Jul. 22, 2014~ Jan. 19, 2015	115,035
Bitcoin	Bitcoin Charts	Sept. 13, 2011~ Jan. 28, 2015	1,233	Bitcoin Forum	Sept. 13, 2011~ Jan. 28, 2015	16,621

#### Experiment 1: EVE Online

The virtual currency unit in EVE Online is called InterStellar Kredit (ISK). The cash transaction of virtual money is based on 2,000,000,000 ISK. In this study, we intended to predict changes in the market price of cash against the applicable reference amount. In the US, many cash transaction services exist for trading ISK currency in US dollars (USD). For data crawling, a tool for integrating and observing those services [77] is used. Specifically, the market prices of ISK from July 22, 2014 through January 22, 2015 (77 data items at an interval of 1~3 days) were analyzed. To collect and analyze opinion data, crawling was performed in the official game forum for EVE Online provided by CPP Games. That is, the forum had 4,587 postings on all discussion topics for 184 days from July 22, 2014 through January 22, 2015. In addition, each topic’s title, posted dates, comment counts, and view counts were likewise saved. We performed the search-engine sentiment analysis, learning, and testing on the crawled opinion data. The result of the Granger casualty test was followed as the relevant virtual world learning that applied sadness and anticipation. The topic examples of the relevant sentiments are as follows in Table 7.

**Table 7:** Sentiment analysis example of EVE Online community.

Emotional Criteria	Example topic sentences
Sadness	“Unfair Advantages” / “This game is like Titanic Movie” / “EVE Launcher not working” / “Game just quit and launcher died.”
Anticipation	“Why Eve isn't more popular?” / “Balancing ships and ammo!” / “T2 Capships coming soon?” / “Maybe Eve isn't dying after all. . . .”

Using the model created above, fluctuation value for 183 days was verified through ten-fold cross-validation (see the results in Table 8; the most accurate model is indicated in bold). To verify the model, the time gap between the opinion data and market price data was set with five combinations from the third day to the seventh day. Given the prediction of fluctuation based on the applicable model, the predictive weighted average precision when the time gap was four days with the anticipation mood was 77.6%, which was the highest value.

**Table 8:** Weighted average precision for EVE Online fluctuation prediction (%).

Lag	Sadness	Anticipation	Sadness and Anticipation
3 days	67.6	70.9	70.9
4 days	71.4	**77.6**	75.3
5 days	52.7	54.7	43.7
6 days	70.5	64.2	72.3
7 days	49.9	55.2	50.1

#### Experiment 2: World of Warcraft

In World of Warcraft, the virtual gaming currency units are called Gold, Silver, and Copper. Gold is the most valuable unit and is traded in cash. A typical Gold cash transaction is based on 50,000 Gold units. As in the EVE Online experiment, we employed a tool [77] for market data crawling. The collected data was the market prices of Gold from July 22, 2014 through January 19, 2015 (74 data items at an interval of 1~3 days). To collect and analyze the opinion data, Blizzard Entertainment’s official game forum [78] was used for crawling. The forum had a total of 115,035 postings on all discussion topics from July 22, 2014 through January 19, 2015 (181 days). In addition, each topic’s title, posted date, comment counts, and view counts were saved. We performed search-engine sentiment analysis, learning, and an evaluation of the crawled opinion data. The result of the Granger casualty test was followed as the relevant virtual world learning applied joy, sadness, and anticipation. The topic examples of the relevant sentiments are as follows in Table 9.

**Table 9:** Sentiment analysis example of World of Warcraft community.

Emotional Criteria	Example topic sentences
Joy	“Thanks, Blizzard!” / “Mists of Pandaria 10 Day Free Trial” / “Anniversary Event Extension” / “Blizzard says it's ok to be a jerk”
Sadness	“Server Maintenance soo. . .rate that xmog!” / “World Server Down” / “The Iron Horde has already failed” / “Sad to see so many leaving”
Anticipation	“The new models aren't final, right?” / “Least/Most popular class?” / “New Race Idea: Gargoyles!” / “Your most memorable WoW moment”

By using the created model, 181 days of market fluctuation data was examined through ten-fold cross-validation (see the results in Table 10; the most accurate model is indicated in bold). Opinion data and market price data were combined based on the time gap. To verify the model, five combinations from the first day to the fifth day were made. As a result, given the prediction of fluctuation based on the applicable model, the weighted average precision when the time gap was two days with the anticipation mood was 69.1%, which was the highest value.

**Table 10:** Weighted average precision for World of Warcraft fluctuation prediction (%).

Lag	Joy	Sadness	Anticipation	Joy and Sadness	Joy and Anticipation	Sadness and Anticipation	Joy, Sadness, and Anticipation
1 day	53	59.9	52.9	55.1	58.5	51.7	51.7
2 days	65.2	61.1	**69.1**	59.9	63.4	63.4	59.9
3 days	52.5	49.2	58.1	50.9	52.1	45.1	56.8
4 days	53.4	44.5	45.5	51	53.5	47.4	45.4
5 days	54.6	50.6	65.7	54.6	60.8	57.5	57.5

The other experiment that we conducted addressed the prediction of fluctuations of Bitcoin. This virtual currency is comparable to those analyzed herein; however, it is not used in virtual worlds. Consequently, it is more relevant to real currencies than to virtual gaming currencies. The cash transaction market for Bitcoin outweighs that for World of Warcraft. Here, we applied to Bitcoin the same algorithm that we applied to Virtual World currencies for prediction. It was assumed that Bitcoin is significant for analysis because transactions with actual money are more frequent than those with virtual money within a game.

#### Experiment 3: Bitcoin

For collection and analysis of Bitcoin-related market data, a service that collects and provides information about worldwide Bitcoin exchanges was used for crawling [79]. Among the many Bitcoin exchanges, Bitstamp, one of the most renowned US Bitcoin exchange markets, was selected. The market data included daily prices from September 13, 2011 through January 28, 2015 (1,233 days). The opinion data about Bitcoin was collected from the largest forum from which the US Bitcoin market trends can be easily accessed [80]. The opinion data included 16,621 postings on all discussion topics during the same period (September 13, 2011 through January 28, 2015). In addition, each topic’s title, posted date, comment counts, and view counts were saved. We performed search-engine sentiment analysis, learning, and testing on the crawled opinion data. The result of the Granger casualty test was followed as the relevant virtual world learning applied trust, fear, and surprise. The topic examples of the relevant sentiments are as follows in Table 11.

**Table 11:** Sentiment analysis example of BitCoin community.

Emotional Criteria	Example topic sentences
Trust	“Forbes Article Predicts Bitcoin Value will "Explode"” / “Good news for the Bitcoin” / “Don't panic, China is NOT banning bitcoin”
Fear	“Mining cartel attack” / “OMG! What has Satoshi created? He has opened Pandora's box” / “We are victims of our own success”
Surprise	“Whatever happened to the Bitcoin Police?” / “I think the rapture happened.....?” / “Blockchain.info "firstbits" changing/disappearing?!”

By applying the model created above, we performed validation to verify the fluctuating value data for 1,095 days (see the results in Table 12; the most accurate model is indicated in bold). The first 85% of the data (1,048 days of observation) were used for model training, while the last 15% (185 days of observation) were used for out-of-sample forecasting. To verify the model, the time gap between the opinion data and market price data was set with seven combinations from the first day to the seventh day. When the time gap was six days with the trust and surprise moods, which produced the best results, the predictive weighted average precision of fluctuation was 76.1% based on the applicable model.

**Table 12:** Weighted average precision for Bitcoin fluctuation prediction (%).

Lag	Trust	Fear	Surprise	Trust and Fear	Trust and Surprise	Fear and Surprise	Trust, Fear, and Surprise
1 day	54.2	58.1	64.6	51.5	39	58	39
2 days	32.8	33	33.1	47.2	41.2	33.1	41.2
3 days	44	41.8	48.8	61.9	54.9	39.1	47.6
4 days	37.1	42.7	36.9	45.1	37.5	37.4	37.5
5 days	49.8	52.9	57.5	54	52.6	54.5	53.6
6 days	55.1	34.3	34.3	75.9	**76.1**	34.3	**76.1**
7 days	61.4	65.8	65.8	63.2	64	52.6	52.6

## Discussion and Conclusion

In this paper, we introduced an analysis system for predicting the value fluctuations of virtual currencies used in virtual worlds based on user opinion data in select online communities. In the proposed method, data of user opinions on a predominant community are collected by employing a simple algorithm and guaranteeing a stable prediction of value fluctuations of more than one virtual currency. With the ever increasing size of the virtual world market size, the proposed prediction of virtual currencies is likely to help developers and users in virtual worlds. In addition, the proposed method was proven to efficiently predict the value of other virtual currencies, including Bitcoin, a non-virtual-world currency; accordingly, the method may be used for purposes beyond the scope of virtual realms. The proposed prediction system provides information on market trends to developers, who can thus numerically and stochastically identify several effects that are expected in planning but difficult to grasp (e.g., user enjoyment, the vitalizing of a virtual economy, easing inflation and deflation, etc.). The proposed prediction system provides virtual currency users with the potential to seek profits by predicting the value fluctuations of virtual currencies, while enabling them broader insights into overall opinions or trends of virtual currencies of interest.

In addition, the proposed sentiment analysis model offers applicability for both developers and users. In this study, the analysis of online communities proved that users’ emotions influence the contents in actual games. This finding suggests that the proposed model enables developers who administer virtual currencies to more easily manage the virtual economy of their own design and to efficiently oversee the interactions among users and virtual currencies by observing the content shifts within games. Furthermore, the proposed sentiment analysis method can be used to grasp overall opinions of internal users in virtual worlds. In short, the proposed method can facilitate virtual world developers’ understandings of user opinions and guide their development activities.

The proposed method needs improvement in a few aspects. First, if investment strategies [73] used for stock forecasting apply the increasing virtual currency assumption, then improved results can be produced. To enhance the precision of sentiment analysis, it is necessary to optimize the analysis in line with virtual world systems. For example, the frequency of words in a virtual community can be investigated, a learning data set for the given virtual world can be developed, and a customized prediction model can be built. In addition, such a data set must not necessarily rely on the opinion data; rather, it should be diversified by adding the components that influence the virtual currency system. For example, virtual currency system updates and changing trends of virtual currency users can be analyzed and added to the data. By improving the system in these ways, the universality and efficiency of the method can be increased.

In sum, we proposed in this paper a simple and universal system for predicting virtual currencies. This system was validated with different virtual currencies to prove its effectiveness in extensively predicting virtual currency fluctuations. In addition, our evaluation of the analysis results shows that the proposed system is significant for both virtual currency developers and users. Because the system was designed for the internal analysis of virtual worlds for general purposes, it is applicable to predicting the values of internal contents of virtual worlds as well as virtual currencies. For example, through the shifts of content values, it is possible to predict the transactions of gaming contents (in-app payments) purchased inside games, not transactions in cash between users. As the influence of virtual currencies on real economies increases, the overall importance of virtual currencies will likewise increase. Hence, the analysis and prediction of virtual currencies may transcend research on a target virtual economy to achieve diversified outcomes in multiple fields, including spot economies and opinion mining. In the near future, the proposed method will likely prove more significant to many fields with more extensive targets and enhanced sentiment analysis.

## Supporting Information

S1 Table Table that displays the result of implementing sentiment analysis from the user opinion data of official game forum for EVE Online provided by CPP Games (https://forums.eveonline.com). (CSV)

S2 Table Table that displays the result of implementing sentiment analysis from the user opinion data of Blizzard Entertainment’s official game forum (http://us.battle.net/wow/en). (CSV)

S3 Table Table that displays the result of implementing sentiment analysis from the user opinion data of the Bitcoin Forum (https://bitcointalk.org). (CSV)
